# The prospective relationship between loneliness, life satisfaction and psychological distress before and during the COVID-19 pandemic in the UK

**DOI:** 10.1007/s10389-022-01719-x

**Published:** 2022-05-27

**Authors:** Jelena Milicev, Pamela Qualter, Claire Goodfellow, Joanna Inchley, Sharon Anne Simpson, Alastair H. Leyland, Kalpa Kharicha, Emily Long

**Affiliations:** 1 MRC / CSO Social and Public Health Sciences Unit, University of Glasgow, Glasgow, G3 7HR UK; 2 Manchester Institute of Education, University of Manchester, Manchester, M13 9PL UK; 3 Campaign to End Loneliness part of What Works Centre for Wellbeing, London, SW1H 9EA UK

**Keywords:** mental health, interventions, neighbourhoods, social ecological model

## Abstract

**Aim:**

Mental wellbeing in the UK seems to have deteriorated significantly during the COVID-19 pandemic, with the rates of loneliness, life satisfaction and psychological distress taking longer to return to the pre-pandemic levels than elsewhere. Nevertheless, there is little knowledge about the interactions between these outcomes, or the factors that played a role in the rates of change. The current study aims to address this gap by simultaneously investigating changes in loneliness, life satisfaction and psychological distress in the UK from pre-pandemic levels to those between April and November 2020, while critically assessing the role of a range of social ecological influencing factors.

**Subject and Methods:**

Longitudinal data from Understanding Society (N=3475) were used to explore the changes in loneliness, life satisfaction and psychological distress from pre-pandemic levels (2017-2019) through November 2020, the interactions between these outcomes, and the role of individual, social, community and geographic factors in the rates of change, using multivariate latent growth curve model.

**Results:**

Loneliness, life satisfaction and psychological distress deteriorated minimally between April and November 2020, compared to the pre-pandemic levels (2017–2019), while the rate of change in each outcome influenced the rates of change in the other two. Key individual (age, gender, physical health), social (number of friends and similarity to them), and environmental (neighbourhood quality) variables influenced baseline scores and the rates of change.

**Conclusion:**

Considering significant dynamic associations between loneliness, life satisfaction and psychological distress, we argue that interventions to tackle any one of the outcomes may have beneficial effects on others, while highlighting malleable factors and individual and community-level interventions to tackle loneliness.

## Introduction

Numerous reports and articles have warned of the potential psychological consequences of the COVID-19 pandemic and the associated quarantine and social distancing measures brought on by governments to contain the spread of the disease (Xiang et al. 2020; Ornell et al. 2020). Nevertheless, there is evidence that after the initial global increase in psychological distress, its prevalence returned to the pre-pandemic levels by mid-2020 (Robinson et al. 2022; Aknin et al. 2022). Furthermore, in some countries, rates of suicide, life satisfaction, and loneliness remained stable, or even improved compared to the pre-pandemic levels or model estimates (Aknin et al. 2022). By contrast, rates of loneliness, life satisfaction and psychological distress in the UK exhibited more negative trends, which also lasted longer (Fujiwara et al. 2020; Office for National Statistics 2021a, b). However, there is little knowledge about how these outcomes interacted over time and which wider factors played a role in the rates of change. The current study addresses this gap by simultaneously investigating changes in loneliness, life satisfaction and psychological distress in the UK from pre-pandemic levels to those between April and November 2020, while critically assessing the role of a range of influencing factors.

### Trends in life satisfaction and psychological distress across the pandemic

Life satisfaction is an individual’s cognitive assessment of their quality of life (Veenhoven 1991), while psychological distress is a unique discomforting, emotional state experienced in response to stressful life events (Ridner 2004). Nationally representative studies showed that in April 2020, a month into the first national lockdown, UK residents reported lower levels of life satisfaction and higher levels of psychological distress compared to the pre-pandemic levels. Risk factors included young age (under 25), female gender, ethnic minority background, low income, having preschool children at home and previous health conditions (Fujiwara et al. 2020; Pierce et al. 2020). Analysing data from the UK Household Longitudinal Study (UKHLS), Daly and Robinson (2021) mapped the next significant rise in psychological distress onto the period of the second COVID-19 wave and the corresponding lockdown measures peaking in January 2021, during which period individuals with school-age children at home were particularly vulnerable. After anxiety and happiness hit the 10-year record high and low, respectively, in the last quarter of 2020, improvements were recorded by April-June 2021, except for the 16-24 year olds, those between 35 and 39, and those over 84 (Office for National Statistics 2021b).

ONS data for the first three quarters of 2020 shows a small, but significant reduction in life satisfaction compared to the pre-pandemic evaluations (Office for National Statistics 2020). Helliwell and colleagues (2021) highlight different trends in the rates of change among different facets of wellbeing in that data set, with anxiety being affected almost twice as much as happiness, and with ratings for these two emotions recovering more quickly than those for life evaluations, i.e. life satisfaction and the sense of purpose. As the lockdown restrictions eased and vaccination in the UK advanced in the period of April – June 2021, the average national levels of life satisfaction returned to those recorded at the start of the pandemic, but, once more, people under 25, those between 35 and 39, and those over 84 years of age did not experience any improvement (Office for National Statistics 2021b).

### Dynamics between loneliness, life satisfaction and psychological distress

Loneliness is a perceived discrepancy between desired and achieved relationships (Perlman and Peplau 1981). It is positively associated with psychological distress (Lim et al. 2020; Loades et al. 2020) and negatively with life satisfaction (Salimi 2011; Bai et al. 2018; Szcześniak et al. 2020). Its link to poor mental and physical health, and mortality (Holt-Lunstad et al. 2015; Holt-Lunstad 2017; O’Súilleabháin et al. 2019) was recognised as a growing public health threat even before the pandemic. From the evolutionary perspective (Cacioppo et al. 2014; Qualter et al. 2015), negative feelings that characterise loneliness serve an adaptive purpose of motivating the person to seek social contact to increase the chances of survival. It has been suggested that lockdowns and social distancing measures introduced to curb the spread of the virus may have thwarted this motivation, thus increasing psychological distress (Luchetti et al. 2020), which is in line with the findings of a longitudinal study from Finnland (Latikka et al. 2022). Rates of loneliness in the UK peaked during the first month of lockdown (March-April 2020), and started to decline in May, returning close to the pre-pandemic levels by June 2020 (Foa et al. 2020). Even though loneliness levels remained relatively stable during the strict lockdown in the UK between March and May 2020, individuals who reported highest levels of loneliness at the beginning of lockdown became even lonelier over time, with risk factors such as younger age (<60), female gender, low household income, being a student, and having a mental health condition potentially increasing the existing inequalities (Bu et al. 2020). While Foa and colleagues (2020) established that during the pandemic, loneliness played a significant role in the life satisfaction of the representative study sample, its role in psychological distress during this period remains unclear.

Considering the well-known correlations between loneliness and psychological distress (Lim et al. 2020), loneliness and life satisfaction (Bai et al. 2018), and psychological distress and life satisfaction (Lombardo et al. 2018), it is important to not only (1) track the changes in each outcome across the pandemic but (2) also understand how changes in one outcome relate to the changes in the other two. Such knowledge is crucial not only if we are to understand how loneliness interventions might work but also why psychological interventions appear to be the most successful at reducing loneliness for people reporting high levels of this feeling, regardless of age (Bessaha et al. 2020; Christiansen et al. 2021; Eccles and Qualter 2021).

The utility of a social ecological perspective (Bronfenbrenner 2005) to understand loneliness among youth (Marquez et al. 2022; Goodfellow et al. 2022b) and adults (Buecker et al. 2021) has been highlighted in recent research. In the current study, we use that same foundation to explore how changes in loneliness, psychological distress and life satisfaction relate to each other, and what role the different social ecological dimensions play in those relationships. We explore the extent to which individual, relational, community and geographic variables predict the rate of change in loneliness, psychological distress and life satisfaction from before the COVID-19 pandemic and during it. Specifically, we use data from *Understanding Society,* the UK Household Longitudinal Study (UKHLS; University Of Essex, ISER 2021) to assess (1) the change in loneliness, life satisfaction and psychological distress over the course of the first 8 months of the COVID-19 pandemic from pre-COVID-19 levels, (2) how changes in those outcomes are related to each other, and (3) whether the rate of change in each outcome was predicted by a key set of social ecological variables.

## Method

### Design and participants

Participants were part of the *Understanding Society* Survey (University Of Essex, ISER 2021), which provides high quality panel data comprising a stratified and clustered general population sample of approximately 40,000 households. The University of Essex Ethics Committee approved all data collection and Informed consent was obtained orally prior to interviews (Institute for Social and Economic Research 2022). Our analyses used data from the 2017–2019 wave (wave 9) and the data collected in April, May, June, July, September and November 2020 during the Covid-19 pandemic (wave 11). The sample, thus, consists of people in the 2017–2019 wave who completed the measurements during the pandemic. Our final analyses include data from 3475 individuals ages 16–94 years from across the UK (see Table 1 for sample information).

**Table 1:** Demographics across current sample

Variable	Number of participants providing those data
Gender *n* (%)	5773
Male = 2411 (41.8%)	
Female = 3362 (58.2%)	
Age	
Mean (SD) = 53 years (15.07)	5773
Range = 16-94 years	
UK Region *n* (%)	
England = 4431 (76.8)	5769
Wales = 385 (6.7%)	
Scotland = 597 (10.3%)	
Northern Ireland = 356 (6.2%)	
NHS Shield *n* (%) = 346 (6.0%)	5773
Neighbourhood quality	5712
Mean (SD) = 2.48 (.734)	
Range = 1-5	
Urban living *n* (%) = 4103 (71.1) Rural living *n* (%) = 1666 (28.9)	5769
Friends of similar age (%)	
Mean (SD) = 2.01 (.970)	
Range = 1-4	5755
Friends of same race (%)	5647
Mean (SD) = 1.62 (.800) Range = 1–4	
Number of close friends	5630
Mean (SD) = 5.62 (4.760) Range = 1–100	
Hours on social media engaged with friends	3608
Mean number of hours = 2.29 (.827)	
Range = 1–5	

## Measures

### Outcome variables

#### Loneliness

Loneliness was measured using the single question asking: ‘How often do you feel lonely?’, with three response categories (1 = ‘hardly ever or never’, 2 = ‘some of the time’ or 3 = ‘often’). The single item of loneliness is recommended by the UK Office of National Statistics as a robust measure of loneliness (Office for National Statistics 2018) and as such was used to assess loneliness at each time point in the study. This is supported by its high correlation with the 3-item loneliness measure (Hughes et al. 2004) also completed by participants at T1 (*r* = .799).

#### Psychological distress

The 12-item General Health Questionnaire (GHQ) was used to measure psychological distress, with higher scores indicating higher distress (Goldberg and Williams 1988). This well-validated scale (Tarnopolsky et al. 1979; Pan and Goldberg 1990; Seva et al. 1992) assesses how respondents’ recent experiences of mental wellbeing compare to their usual functioning with the aim of identifying psychological distress and potential cases of common psychiatric disorders. Items include depressive and anxiety symptoms, sleeping problems and overall happiness. Understanding Society (Institute for Social and Economic Research 2022) converts the answers to GHQ-12 questions to a single continuous scale ranging from 0 (the least distressed) to 12 (the most distressed).

#### Life satisfaction

This was measured by a single item asking: ‘Overall, how satisfied are you with your life nowadays?’. Response categories range from 1 (completely unsatisfied) to 7 (completely satisfied) (Dolan et al. 2011).

### Predictor variables

#### Individual characteristics

We included several individual difference variables in our analyses. In line with previous literature (Claramonte Nieto et al. 2019), to explore the impact of *age* on the baseline and change in loneliness, psychological distress and life satisfaction, we created two categorical variables using participant age: younger adults (0 = 16–24 years of age; 1 = over 25 years of age), and older adults (0 = 65 years of age or over; 1 = 64 years or younger). *Sex* was a binary variable (0 = male; 1 = female), as was whether the participant had a *medical condition* that required them to shield during the pandemic (0 = No; 1 = Yes), and whether their relationship status was *single* or not (1 = not single, 0 = single).

#### Social relationships

Participants completed several measures about their friendships at baseline. First, they provided information on whether their friends were of a similar age or ethnicity to them, using the following scale: 1 = all similar, 2 = more than half, 3 = about half, 4 = less than half. They also noted the number of close friends they had, and the number of hours per day they spent interacting with those friends on social media (1 = none, 2 = less than an hour, 3 = 1–3 hours, 4 = 4–6 hours, 5 = 7 or more hours).

#### Community

Perceived neighbourhood quality was measured using a mean score from 4 separate variables: “belonging to the neighbourhood”, “being similar to others in the neighbourhood”, “talking regularly with neighbours”, and “having local friends”. Participants answered using a 5-point scale, which were all reverse coded so that higher scores representing worse neighbourhood quality. Participants also noted whether they lived in an urban or rural area (0 = rural, 1= urban).

#### Geographic region

We used broad geographic region in our analyses, noting whether participants were residents in England, Wales, Scotland or Northern Ireland. For inclusion in our analyses, we created a binary variable for each country, with the country as the ‘target group’ (coded as ‘1’) and the other nations combined to create our ‘comparison group, which we coded as ‘0’.

### Analyses plan

Longitudinal data on life satisfaction, loneliness and self-reported psychological distress were analysed using a multivariate latent growth curve model (MLGC) in Mplus. Our MLGC model was a single model of growth in the three outcomes, where we fit the three simultaneous growth curves and estimate covariances among their growth factors. We used linear growth models with continuous outcomes; models were estimated using the robust maximum likelihood (MLR) estimator, recommended as the preferred method for accounting for missing data (Graham 2012).

Model fit was evaluated using root mean square error of approximation (RMSEA), comparative fit index (CFI) and standardised root mean square residual (SRMR). RMSEA values of less than 0.05 indicate a close fit, values up to 0.08 represent reasonable errors of approximation, and CFI values > 0.95 represent good fit (Little 2013); we used a cut-off value of < 0.09 for the SRMR (Cho et al. 2020). Variances in the model were also explored to determine whether there was justification to incorporate predictor variables into subsequent analyses to explain the parameter estimates.

In the first model (model A) we explored the growth of life satisfaction, loneliness and self-reported psychological distress from baseline (before the COVID-19 pandemic) across the six time points during the COVID-19 pandemic in the UK (April–November 2020). We also explored whether there was variance in the initial model, which would suggest differences both in the initial status and change in our variables over time; such variance would support incorporating predictor variables (demographics) into subsequent analyses (model B). Data met the criteria for using MLGC, so we conducted analyses for model A and B. We used a *p* < 0.05.

## Results

Model A showed a reasonable fit to the data (RMSEA = .076 [.074, .078], CFI = .890, SRMR = .059). There were improvements to model fit for model B, with the inclusion of demographic and individual differences variables into the model (RMSEA = .060 [.059, .062], CFI = .877, SRMR = .037). Table 2 includes the estimated means of loneliness, life satisfaction and psychological distress, demonstrating minimal fluctuation from pre-COVID-19 levels and then during the first 8 months of the COVID-19 pandemic in the UK, which included several national and local lockdowns (Institute for Government 2021). Table 2 includes the findings for model B. It shows high variability in psychological distress at every time point, and important associations between loneliness and psychological distress (*rs* = .478 [T1] to .591 [T7]), loneliness and life satisfaction (*r*s = -.293 [T5] to -.383 [T1]), and life satisfaction and psychological distress (*r*s = -.364 [T7] to -.510 [ T1]).

**Table 2:** Estimated sample statistics and model results for model B

Estimated means																													
				2017-2019 wave		April 2020				May 2020			June 2020			July 2020			September 2020			November 2020							
				**T1**		**T2**				**T3**			**T4**			**T5**			**T6**			**T7**							
Loneliness				1.375		1.391				1.380			1.367			1.360			1.371			1.427							
Life satisfaction				5.285		–				–			–			5.055			4.972			4.975							
Psychological distress				10.931		12.039				11.961			12.028			11.407			11.627			12.409							
Covariances																													
**Loneliness**														**Life satisfaction**									**Psychological distress**						
Loneliness			T1		T2	T3		T4	T5		T6	T7		T1		T2	T3	T4	T5		T6	T7	T1	T2	T3	T4	T5	T6	T7
		T1	0.368											–0.328															
		T2	0.153		0.363									–0.207		–							1.000						
		T3	0.157		0.232	0.342								–0.225		–	–						1.073	1.745					
		T4	0.158		0.214	0.234		0.326						–0.230		–	–	–					1.099	1.680	1.791				
		T5	0.156		0.200	0.215		0.228	0.317					–0.240		–	–	–					1.110	1.625	1.721	1.802			
		T6	0.167		0.194	0.208		0.213	0.223		0.336			–0.241		–	–	–	–0.238				1.178	1.582	1.673	1.715	1.665		
		T7	0.168		0.200	0.213		0.215	0.219		0.233	0.353		–0.253		–	–	–	–0.244		–0.268		1.153	1.610	1.693	1.715	1.579	1.644	
Life satisfaction		T1												1.993															
		T2			–									–		–								–	–				
		T3			–	––								–		–	–							–	–	–			
		T4			–	––		–						–		–	–	–						–	–	–			
		T5	-0.182		–0.185	–0.200		–0.221	–0.255					0.674					2.399				–1.906	–2.404	–2.715	–2.808			
		T6	-0.226		–0.206	–0.229		–0.246	–0.263		–0.303			0.654					0.971		2.216		–2 .022	–2.706	–2.897	–3.025	–3.004		
		T7	-0.177		–0.186	–0.208		–0.218	–0.238		–0.229	–0.263		0.644					0.882		0.906	2.268	–1.918	–2.466	–2.558	–2.594	–2.549	–2.652	
Psychological distress		T1	1.534											–3.805									27.947						
		T2	1.191		1.925									–2.515									13.864	34.291					
		T3	1.189		1.636	1.939								–2.546									14.369	25.066	32.834				
		T4	1.163		1.456	1.642		1.911						–2.505									14.296	22.861	25.205	34.154			
		T5	1.137		1.287	1.420		1.551	1.806					–2.567					–3.232				14.240	20.191	21.977	24.160	30.206		
		T6	1.186		1.279	1.321		1.432	1.536		1.869			–2.490					–2.630		–3.536		13.930	18.875	19.797	21.696	21.515	30.353	
		T7	1.210		1.340	1.380		1.479	1.540		1.668	2.041		–2.529					–2.526		–3.020	–3.183	14.299	19.663	20.316	21.428	20.784	22.165	33.733
Correlations																													
Loneliness									Life satisfaction							Psychological distress													
Loneliness		T1	T2	T3	T4	T5	T6	T7	T1	T2	T3	T4	T5	T6	T7	T1	T2	T3	T4	T5	T6	T7							
	T1	1.000							–0.383																				
	T2	0.419	1.000						–0.243							0.314													
	T3	0.442	0.658	1.000					–0.272							0.347	0.510												
	T4	0.457	0. 622	0.702	1.000				–0.285							0.364	0.503	0.548											
	T5	0.458	0. 589	0.655	0.710	1.000			–0.302							0.373	0.493	0.534	0.548										
	T6	0.474	0. 554	0.612	0.645	0.683	1.000		–0.295				–0.265			0.384	0.466	0.503	0.506	0.522									
	T7	0.467	0.559	0.612	0.633	0.654	0.677	1.000	–0.302				–0.265	–0.303		0.367	0.463	0.497	0.494	0.483	0.502								
Life satisfaction	T1								1.000																				
	T2																												
	T3																												
	T4																												
	T5	–0.194	–0.198	–0.221	–0.250	–0.293			0.308				1.000			–0.233	–0.265	–0.306	–0.310										
	T6	–0.251	–0. 230	–0.263	–0.290	–0.314	–0.351		0.311				0.421	1.000		–0.257	–0.310	–0.340	–0.348	–0.367									
	T7	–0.194	–0.205	–.236	–0.254	–0.281	–0.262	–0.294	0.303				0.378	0.404	1.000	–0.241	–0.280	–0.296	–0.295	–0.308	–0.320								
Psychological distress	T1	0.478							–0.510							1.000													
	T2	0.335	0.545						–0.304							0.448	1.000												
	T3	0.342	0.474	0.579					–0.315							0.474	0.747	1.000											
	T4	0.328	0.413	0.481	0.573				–0.304							0.463	0.668	0.753	1.000										
	T5	0.341	0.389	0.442	0.495	0.584			–0.331				–0.380			0.490	0.627	0.698	0.752	1.000									
	T6	0.355	0.385	0.410	0.455	0.495	0.585		–0.320				–0.308	–0.431		0.478	0.585	0.627	0.674	0.711	1.000								
	T7	0.343	0.383	0.407	0.446	0.471	0.495	0.591	–0.308				–0.281	–0.349	–0.364	0.466	0.578	0.610	0.631	0.651	0.693	1.000							

Exploration of the intercepts and slopes from model B showed small but significant increases in loneliness from pre-pandemic levels, reductions in life satisfaction and increases in psychological distress (Table 3). There was evidence that the rate of change of loneliness and life-satisfaction for those with higher psychological distress at baseline was somewhat slower, while no other relationships between baseline scores and rates of change were significant (Table 4). Furthermore, the rates of change in the different outcomes were associated, as per slopes in Table 4, with those following an increasing trajectory of loneliness showing an associated decrease in life satisfaction and increase in psychological distress, which, in turn, was also associated with a decrease in life satisfaction.

**Table 3:** Intercept and slope mean parameter estimates

	Estimate	Standard error (*SE*)	Estimate/*SE*	*p*
Intercept loneliness	1.368	.007	201.296	<.001
Slope loneliness	.004	.001	3.574	<.001
Intercept life satisfaction	5.278	.018	287.286	<.001
Slope life satisfaction	–.055	.004	-15.305	<.001
Intercept psychological distress	11.548	.069	168.074	<.001
Slope psychological distress	.076	.011	6.771	<.001

**Table 4:** Parameter estimates for model B

Model results				
	Estimate	Standard error (*SE*)	Estimate/*SE*	*p*-value
Intercept of loneliness WITH				
Slope of loneliness	.028	.066	.428	.668
Intercept of life satisfaction WITH				
Slope of life satisfaction	.086	.315	.273	.785
Intercept of loneliness	–.611	.073	–8.391	< .001
Slope of loneliness	.006	.077	.075	.940
Intercept of psychological distress WITH				
Slope of psychological distress	–.011	.073	–.0148	.882
Intercept of loneliness	.815	.019	43.560	<.001
Slope of loneliness	–.158	.054	–2.897	.004
Intercept for life satisfaction	–.815	.092	–8.865	<.001
Slope of life satisfaction	.174	.066	2.620	.009
Slope of life satisfaction WITH				
Intercept of loneliness	.068	.060	1.117	.264
Slope of loneliness	–.670	.170	–3.933	<.001
Slope of psychological distress WITH				
Intercept of loneliness	–.0447	.061	–7.311	<.001
Slope of loneliness	1.784	.194	9.183	<.001
Intercept of life satisfaction	.356	.081	4.370	<.001
Slope of life satisfaction	–1.191	.261	–4.558	<.001

Specific demographics and individual differences predicted life satisfaction, loneliness and self-reported psychological distress at baseline, and the rate of change in those outcomes during the pandemic. The following were significant predictors of loneliness at baseline, pre-COVID-19 (Table 5): being under 25, compared to those aged 25 or over (﻿β=-0.140, SE=0.023, *p*<0.001) or over 65 years of age, compared to those under 65 (﻿β=-0.060, SE=0.022, *p*=0.006), being female (β=0.069, SE=0.017, *p*<0.001), having a medical condition that required shielding (﻿β=0.193, SE=0.019, *p*<0.001), having few friends of the same age (﻿β=0.051, SE=0.021, *p*=0.014) and ethnicity (﻿β=0.061, SE=0.022, *p*=0.005), and living in a poor-quality neighbourhood (﻿β=0.208, SE=0.022, *p*<0.001). Having more close friends (﻿β=-0.056, SE=0.021, *p*=0.006) and being in a relationship (﻿β=-0.087, SE=0.023, *p*<0.001), as opposed to being single, were associated with less loneliness. ﻿Life satisfaction at baseline was predicted by age, with adults over 25 (﻿β=0.374, SE=0.041, *p*<0.001) and younger than 65 years (﻿β=0.080, SE=0.028, *p*=0.004), reporting higher life satisfaction, as well as the people with a higher number of close friends (﻿β=0.096, SE=0.026, *p*<0.001). Females (﻿β=-0.133, SE=0.028, *p*<0.001), those with fewer friends of similar age (﻿β=-0.079, SE=0.027, *p*=0.004)﻿and those living in poorer quality neighbourhoods ﻿(β=-0.245, SE=0.033, *p*<0.001) reported lower life satisfaction. Psychological distress at baseline was lower for those 25 and older (﻿β=-0.164, SE=0.024, *p*<0.001), compared to 16-24 year-olds, and for those under 65 (﻿β=-0.048, SE=0.021, *p*=0.025), compared to those aged 65 and older. It was also lower for those in a relationship (﻿β=-0.047, SE=0.021, *p*=0.023), compared to singles, and those with more close friends (﻿β=-0.051, SE=0.022, *p*=0.020). It was higher for females (﻿β=0.110, SE=0.017, *p*<0.001), individuals required to shield during COVID-19 (﻿β=0.179, SE=0.018, *p*<0.001), those from poor neighbourhoods (﻿β=0.145, SE=0.022, *p*<0.001), and those with few friends of the same age (﻿β=0.041, SE=0.020, *p*=0.042) and ethnicity (﻿β=0.040, SE=0.020, *p*=0.047).

**Table 5:** Predictors in model B

Model results				
	Estimate	Standard error (*SE*)	Estimate/*SE*	*p*-value
Loneliness				
25+ age group (ref. 16–24 years)	–0.140	0.023	–5.989	0.000
<65 age group (ref. 65+ years)	–0.060	0.022	–2.727	0.006
F gender (ref. M)	0.069	0.017	4.067	0.000
Relationship (ref. Single)	–0.087	0.023	–3.864	0.000
NHS shielded	0.193	0.019	10.438	0.000
Friends of similar age	0.051	0.021	2.467	0.014
Friends of similar ethnicity	0.061	0.022	2.804	0.005
Number of close friends	–0.056	0.021	–2.735	0.006
Amount of time on social media with friends	–0.029	0.023	–1.286	0.198
Neighbourhood quality	0.208	0.022	9.568	0.000
England	0.216	0.128	1.688	0.091
Wales	0.144	0.077	1.871	0.061
Scotland	0.181	0.092	1.967	0.049
Northern Ireland	0.149	0.077	1.944	0.052
Urban	–0.010	0.020	–0.516	0.606
Change in loneliness				
25+ age group (ref. 16–24 years)	0.009	0.039	0.222	0.824
<65 age group (ref. 65+ years)	0.030	0.043	0.684	0.494
F gender (ref. M)	–0.007	–0.034	–0.203	0.839
﻿Relationship (ref. Single)	0.011	0.044	0.255	0.798
NHS shielded	–0.091	0.037	–2.424	0.015
Friends of similar age	–0.008	0.041	–0.187	0.851
Friends of similar ethnicity	–0.017	0.043	–0.406	0.685
Number of close friends	0.086	0.037	2.320	0.020
Amount of time on social media with friends	0.029	0.035	0.825	0.409
Neighbourhood Quality	–0.047	0.044	–1.078	0.281
England	0.020	0.218	0.093	0.926
Wales	0.013	0.134	0.099	0.921
Scotland	–0.102	0.157	–0.648	0.517
Northern Ireland	0.014	0.125	0.112	0.911
Urban	0.006	0.039	0.145	0.885
Life satisfaction				
25+ age group (ref. 16–24 years)	0.374	0.041	9.144	0.000
﻿﻿﻿ <65 age group (ref. 65+ years)	0.080	0.028	2.906	0.004
F gender (ref. M)	–0.133	0.028	–4.701	0.000
﻿Relationship (ref. Single)	0.017	0.026	0.634	0.526
NHS shielded	0.015	0.026	0.586	0.558
Friends of similar age	–0.079	0.027	–2.862	0.004
Friends of similar ethnicity	0.022	0.029	0.753	0.452
Number of close friends	0.096	0.026	3.661	0.000
Amount of time on social media with friends	–0.070	0.045	–1.558	0.119
Neighbourhood quality	–0.245	0.033	–7.387	0.000
England	0.420	0.331	1.266	0.206
Wales	0.266	0.196	1.356	0.175
Scotland	0.242	0.237	1.019	0.308
Northern Ireland	0.254	0.199	1.274	0.203
Urban	0.000	0.027	–0.010	0.992
Change in life satisfaction				
25+ age group (ref. 16–24 years)	–0.211	0.055	–3.839	0.000
<65 age group (ref. 65+ years)	–0.037	0.043	–0.861	0.389
F gender (ref. M)	0.158	0.050	3.172	0.002
﻿Relationship (ref. Single)	0.030	0.040	0.738	0.460
NHS shielded	–0.037	0.041	–.0917	0.359
Friends of similar age	0.023	0.042	0.558	0.577
Friends of similar ethnicity	–0.091	0.050	–1.809	0.070
Number of close friends	–0.098	0.040	–2.456	0.014
Amount of time on social media with friends	0.048	0.070	0.684	0.494
Neighbourhood quality	0.118	0.047	2.532	0.011
England	–0.986	0.595	–1.657	0.097
Wales	–0.610	0.353	–1.729	0.084
Scotland	–0.659	0.424	–1.554	0.120
Northern Ireland	–0.571	0.357	–1.599	0.110
Urban	0.043	0.044	0.986	0.324
Psychological distress				
25+ age group (ref. 16–24 years)	–0.164	0.024	–6.740	0.000
<65 age group (ref. 65+ years)	–0.048	0.021	–2.242	0.025
F gender (ref. M)	0.110	0.017	6.327	0.000
﻿Relationship (ref. Single)	–0.047	0.021	–2.268	0.023
NHS shielded	0.179	0.018	9.799	0.000
Friends of similar age	0.041	0.020	2.032	0.042
Friends of similar ethnicity	0.040	0.020	1.983	0.047
Number of close friends	–0.051	0.022	–2.323	0.020
Amount of time on social media with friends	0.008	0.028	0.296	0.768
Neighbourhood quality	0.145	0.022	6.722	0.000
England	0.009	0.150	0.061	0.952
Wales	0.012	0.089	0.136	0.892
Scotland	0.040	0.108	0.371	0.711
Northern Ireland	0.028	0.090	0.310	0.757
Urban	–0.013	0.020	–0.676	0.499
Change in psychological distress				
25+ age group (ref. 16–24 years)	0.043	0.051	0.849	0.396
<65 age group (ref. 65+ years)	0.039	0.040	0.976	0.329
F gender (ref. M)	–0.040	0.028	–1.455	0.146
﻿Relationship (ref. Single)	0.004	0.039	0.092	0.927
NHS shielded	–0.044	0.035	–1.236	0.216
Friends of similar age	0.032	0.038	0.851	0.395
Friends of similar ethnicity	0.022	0.039	0.558	0.577
Number of close friends	0.021	0.048	0.432	0.666
Amount of time on social media with friends	–0.044	0.038	–1.147	0.251
Neighbourhood Quality	0.025	0.042	0.611	0.541
England	0.179	0.246	0.730	0.465
Wales	0.100	0.147	0.684	0.494
Scotland	0.060	0.178	0.337	0.736
Northern Ireland	0.086	0.146	0.588	0.556
Urban	–0.016	0.036	–0.441	0.659

Change in loneliness from baseline and over the first 8 months of the COVID-19 pandemic was predicted by the following variables collected at baseline: (1) whether the individual had a medical condition requiring shielding (﻿β=-0.091, SE=0.037, *p*=0.015), with those with such a medical condition becoming lonelier at a slower rate over the course of the pandemic; and (2) the reported number of close friends, with loneliness increasing faster in those with more friends at baseline (﻿β=0.086, SE=0.037, *p*=0.020). We also found that life satisfaction reduced more slowly in those over 25, compared to the younger age group (16–24 years) (﻿β=-0.211, SE=0.055, *p*<0.001), as was the case for those with a higher number of close friends (﻿β=-0.098, SE=0.040, *p*=0.014). Females (﻿β=0.158, SE=0.050, *p*=0.002) and those who reported living in poor quality neighbourhoods (﻿β=0.188, SE=0.047, *p*=0.011) experienced a faster reduction of life satisfaction, compared to males and those living in better quality neighbourhoods. Geographic region, or the distinction between urban and rural areas, did not play a role in any of the outcomes. Change in psychological distress was not predicted by any of our predictor variables.

## Discussion

Using data from a large population study where responses were collected pre-COVID-19 pandemic and during the first 8 months of the pandemic and associated lockdowns in the UK, we explored (1) fluctuations in the reported levels of life satisfaction, psychological distress and loneliness; (2) whether changes in any one of these outcomes were influenced by changes in the remaining two; and (3) the extent to which individual, relational, community, and geographic variables predicted the rate of change in the said outcomes. Compared to 2017–2019, we detected an increase in loneliness and psychological distress and a decrease in life satisfaction between April and November 2020, but the changes were minimal. In addition, we found an association in the rates of change in the three outcomes, as those who felt increasingly lonely over the first 8 months of the pandemic experienced an associated increase in distress levels and decrease in life satisfaction. Using the social ecological model, we identified several pre-pandemic risk factors for loneliness and psychological distress, including young age (16-24 years) and being 65 years of age or older, female gender, single relationship status, having a medical condition that required shielding during the pandemic, lower neighbourhood quality, smaller number of friends of the same age and ethnicity, and smaller number of close friends. Protective factors for life satisfaction at baseline were being between 25 and 65 years of age, male gender, better quality neighbourhoods, having more close friends and more friends of similar age. Finally, while loneliness was higher for those with a medical condition before the pandemic, during the pandemic, loneliness increased more slowly for these individuals than those without a medical condition that required shielding. Loneliness grew faster in those with more close friends at baseline. Slower decrease of life satisfaction was reported by those aged 25 and over, and those with more friends; females and individuals from poor quality neighbourhoods experienced an accelerated deterioration of this wellbeing metric.

Our findings are in line with cross-sectional studies showing significant associations between psychological distress, life satisfaction and loneliness (Salimi 2011; Bai et al. 2018; Lim et al. 2020; Loades et al. 2020; Szcześniak et al. 2020). They are consistent with recent work identifying a small deterioration in the population wellbeing during the pandemic (Robinson et al. 2022; Aknin et al. 2022), and with the studies highlighting risk factors such as young and older age and female gender (Fujiwara et al. 2020; Rossi et al. 2020; Pierce et al. 2020). Our findings on the role of neighbourhood quality support the well-evidenced negative association with loneliness and mental distress (Scharf et al. 2005; Diez Roux and Mair 2010), as well as the protective role of neighbourly behaviours and familiarity (Kearns et al. 2015; Goodfellow et al. 2022a).

The current study is the first to explore associations in the rates of change in psychological distress, life satisfaction and loneliness over time during the pandemic. Work has shown relationships between these variables in cross-sectional research, and us using multivariate latent growth curve models to explore the simultaneous changes in the three outcomes, we found that individuals who were experiencing higher levels of psychological distress prior to the pandemic experienced slower changes in life satisfaction and loneliness, while the changes in the three outcomes were mutually associated during the first 8 months of the pandemic. Such findings add to the debate about cognitive life evaluations, e.g. life satisfaction, as being more stable than ratings of emotions (Eid and Diener 2004), which form the basis of the GHQ-12 questionnaire, used to measure psychological distress. As such, our findings offer new insight into the relationships between loneliness, life satisfaction and psychological distress, adding to the body of theoretical work into the mechanisms underpinning this interrelatedness (Qualter et al. 2015; de Gierveld et al. 2018).

This is the first study to identify individual differences that contribute to the rate of change in mental health outcomes during the pandemic. Faster increases in loneliness among people with more close friends at baseline could be underpinned by higher sociability of these individuals, which enabled them to have more close friends, but also made them less tolerable to the enforced lack of contact. While having a medical condition that required shielding was associated with becoming lonelier at a slower rate, it was unrelated to the changes in psychological distress, which is in line with recent findings from Israel (Palgi et al. 2020). This lack of association could be explained by greater experience of being alone and dealing with challenging medical situations, which may have contributed to this group’s resilience.

Females and those under 25 years of age in our study also experienced higher psychological distress and loneliness. This is in line with the evidence of female gender as a risk factor for psychological distress (Matud et al. 2015; Van Droogenbroeck et al. 2018; Zhang et al. 2018). Faster deterioration in life satisfaction among women is in line with the cross-sectional findings highlighting female gender as a risk factor for life satisfaction correlates such as loneliness and psychological distress during the pandemic (Fujiwara et al. 2020; Pierce et al. 2020). Pre-pandemic longitudinal studies showed that life satisfaction of women in the UK was positively associated with employment and negatively with childcare and unpaid care work (Della Giusta et al. 2011). The accelerated decrease in women’s life satisfaction in the pandemic may therefore be related to the evidence of women bearing most of the burden of the increased childcare and unpaid care work in the pandemic (Sevilla and Smith 2020; Power 2020), while facing adverse employment outcomes, including the increased risk of unemployment, compared to men in this situation (Petts et al. 2021).

Close social ties and broader community played a significant role in baseline loneliness and life satisfaction and in their rates of change. The protective effect of having friends of similar age and a higher number of close friends is in line with the existing literature on the positive role of friends and social integration in the wellbeing during life crises (Bolger and Eckenrode 1991; Hintikka et al. 2000; Landberg and Recksiedler 2018). By contrast, low quality neighbourhood as a risk factor may be linked to the experience of ‘double jeopardy’ – dealing with personal poverty, as well as living in an area that offers limited opportunities for a healthy life (Ribeiro 2018). Disadvantaged neighbourhoods have been associated with overcrowding, limited access to health care and green spaces, higher incidence of chronic conditions, and unhealthy coping behaviours, such as alcohol and smoking. These factors may have contributed to higher vulnerability to the virus and longer recovery times (Sharifi 2021), while the ‘stay at home’ orders took away the ability to travel out of these disadvantaged areas, potentially exacerbating their negative impact and affecting the way residents evaluated their lives. Importantly, unlike individual socio-demographic factors such as age and gender, both number of close friends and the perceived neighbourhood quality are malleable factors, and therefore suitable targets for preventative efforts and public health interventions.

While the number of friends and neighbourhood perception were linked to loneliness, the changes in this outcome were closely related to those in life satisfaction and psychological distress. This implies that interventions to target loneliness might simultaneously improve the other two outcomes. Loneliness is linked not only to poor life satisfaction but also to a whole host of physical and psychiatric issues and psychosocial risk factors, including social anxiety, depression, unemployment, alcoholism, suicidal ideation and premature mortality (Holt-Lunstad et al. 2015; Cacioppo et al. 2015). Its strong association with negative social cognitions and poor coping strategies, such as withdrawing and obsessing about problems (Vanhalst et al. 2015; Matthews et al. 2019) contributes to its self-perpetuating nature, increasing the burden on the individual and on public health over time and indicating the need for early interventions to tackle loneliness and the associated problems. Recent evidence (Christiansen et al. 2021; Hickin et al. 2021) demonstrates the effectiveness of cognitive behavioural approaches in targeting maladaptive thoughts and behaviours that underpin loneliness. Their success is believed to lie with the targeting of the maladaptive thoughts and behaviours that underlie both loneliness and mental distress. Changing these thoughts alleviates the underlying anxieties, facilitating positive changes in social behaviours and resulting in reduction in loneliness over time (Qualter et al. 2015; Mann et al. 2017). This joint effect on psychological distress and loneliness is particularly important considering the widely evidenced associations between them (Lim et al. 2020; Loades et al. 2020), confirmed by our findings.

Nevertheless, Mann and colleagues (2017) warn that targeting individual maladaptive thoughts may not be sufficient if the wider context in which the individual exists is not considered. In this sense, they argue that general connectedness in the community can boost individuals’ confidence as community members and facilitate their better integration. They stress the potential of community-based interventions such as (a) social prescribing, which aims to reduce loneliness by connecting people to the local sources of support﻿ (2017), and (b) asset-based community development, whereby individuals are encouraged to create their own community groups and projects, which improve local connectedness, as well as sustainability (Bickerdike et al. 2017; Moffatt et al. 2017; Wildman et al. 2019; Kellezi et al. 2019; Foster et al. 2021). Given the prominent role of neighbourhood quality in our study, our findings support community-based approaches to tackle loneliness, especially interventions that encourage neighbourly behaviours and familiarity within low quality neighbourhoods, which may moderate the negative effect of loneliness on life satisfaction and psychological distress (Kearns et al. 2015; Goodfellow et al. 2022a). We, therefore, highlight the importance of providing guidance and support for safe social interactions through the varying levels of risk during the pandemic and as we emerge from it.

Our findings directly inform interventions to tackle loneliness and are therefore important for policymakers and governments, although continued examination of the psychological impact of the COVID-19 pandemic, beyond the acute phase(s) is essential. Given that changes in psychological distress varied within the population, we recommend that certain groups are monitored during the pandemic and through the gradual return to unrestricted social contact. While we highlight risk groups for increasing loneliness (those with more close friends) and decreasing life satisfaction (females and those in poor quality neighbourhoods), we found that none of our predictor variables contributed to a faster deterioration of psychological distress. Future work should explore other potential predictors of change in psychological distress and other facets of wellbeing.

## Limitations of this study

The current study is not without limitations. First, analyses were performed with a limited number of predictors available in the survey, preventing us from exploring other variables that could be expected to impact the outcomes. Second, all included variables were based on participant self-reports, potentially limiting the validity of measurement due to question misinterpretation or a variety of known biases, including recall, acquiescence, nonresponse and social desirability bias (Hunger et al. 2013; Caputo and Caputo 2017). Finally, although the survey sample is a probability sample, not all sections of the population were selected with the same probability and not everyone asked to participate agreed to it (Institute for Social and Economic Research 2022). This may have created sampling bias, implying that caution is needed in generalising the findings outside of the study population.

## Conclusions

Using longitudinal *Understanding Society* data, the current study contributes new knowledge about the dynamic interactions between psychological distress, life satisfaction and loneliness during the COVID-19 pandemic in the UK. We found an association in the rates of change in the three outcomes, as those whose distress levels were increasing also experienced an associated decrease in life satisfaction and an increase in loneliness. Using the social ecological model, we identified female gender, young and older age, low number of close friends and poor-quality neighbourhoods as some of the risk factors for baseline psychological distress, life satisfaction, and loneliness. Older age and higher number of friends, in turn, protected individuals from decreasing life satisfaction, while being a female or living in a low-quality neighbourhood was linked to accelerated decrease in life satisfaction. Finally, having a medical condition that required shielding and fewer close friends contributed to slower changes in the experience of loneliness. We highlight the number of close friends and perceived neighbourhood quality as malleable factors that could be targeted in public health interventions, as well as individual and community-level interventions to tackle loneliness.

## Data Availability

All Understanding Society Survey (University Of Essex, ISER 2021) data are deposited with UK Data Service repository. 10.5255/UKDA-SN-6614-16

## Code availability

Code will be made publicly available through the Open Science Framework website https://osf.io
