# Correction to: The prospective relationship between loneliness, life satisfaction and psychological distress before and during the COVID-19 pandemic in the UK

**DOI:** 10.1007/s10389-022-01723-1

**Published:** 2022-06-08

**Authors:** Jelena Milicev, Pamela Qualter, Claire Goodfellow, Joanna Inchley, Sharon Anne Simpson, Alastair H. Leyland, Kalpa Kharicha, Emily Long

**Affiliations:** 1 MRC / CSO Social and Public Health Sciences Unit, University of Glasgow, Glasgow, G3 7HR UK; 2 Manchester Institute of Education, University of Manchester, Manchester, M13 9PL UK; 3 Campaign to End Loneliness part of What Works Centre for Wellbeing, London, SW1H 9EA UK

**Correction to: Journal of Public Health**

10.1007/s10389-022-01719-x

The authors found a small error in the concluding paragraph of the manuscript. The paper incorrectly states ‘Young age’, rather than ‘Older age’.

Incorrect sentence (should read ‘Older age’):

Young age and higher number of friends, in turn, protected individuals from decreasing life satisfaction, while being a female or living in a low-quality neighbourhood was linked to accelerated decrease in life satisfaction.

The original article has been corrected.

